# Hydroquinidine as rescue therapy of arrhythmic storm in ischemic cardiomyopathy with severely impaired left ventricular ejection fraction

**DOI:** 10.1016/j.hrcr.2024.07.008

**Published:** 2024-07-14

**Authors:** Thomas Hanquiez, Alexis Hermida, Christophe Beyls, Cedric Renard, Maciej Kubala

**Affiliations:** ∗ Department of Cardiology, Amiens University Hospital, Amiens, France; † EA 7517, Jules Verne University of Picardie, Amiens, France; ‡ Department of Anesthesiology and Critical Care Medicine, Amiens University Hospital, Amiens, France; § 7518 SSPC (Simplification of Care of Complex Surgical Patients) Research Unit, Jules Verne University of Picardie, Amiens, France

**Keywords:** Ventricular tachycardia, Hydroquinidine, Arrhythmic storm, Ischemic cardiomyopathy, Short-coupled premature ventricular contractions, Purkinje system

**Key Teaching Points**

• Hydroquinidine can be used for ventricular electrical storm with efficacy in patients with Brugada syndrome, early repolarization syndrome, short QT syndrome. and idiopathic ventricular fibrillation. Because of its potential proarrhythmic effects, hydroquinidine is often avoided in patients with organic heart disease and there are few data on its use in cardiomyopathies. The effectiveness of hydroquinidine in suppressing drug-refractory polymorphic events starting with short-coupled premature ventricular contractions has been reported during the recovery phase of myocardial infarction in patients with mildly impaired left ventricular ejection fraction (LVEF).
• Recurrent polymorphic ventricular tachycardias triggered by short-coupled premature ventricular contractions (PVCs) during the healing phase following myocardial infarction presented here were refractory to conventional medical therapy and showed no response to deep sedation, cardiac pacing, and stellate ganglion blockade and were contraindicated to catheter ablation because of an intraventricular thrombus. This case demonstrates that hydroquinidine can be used as rescue therapy of arrhythmic storm in ischemic cardiomyopathy with severely impaired LVEF.
• The mechanism of action of hydroquinidine in patients with short-coupled PVCs from damaged Purkinje system remains to be elucidated. This therapeutic option can be taken into account in similar clinical situations. Potential side effects as negative inotropic effect, QT prolongation, and the risk of inducing bradycardia while associated with antiarrhythmic agents should be kept in mind.

## Introduction

Hydroquinidine is largely accepted as the antiarrhythmic drug of choice for preventing recurrence of polymorphic ventricular tachycardia (VT) in patients without structural heart disease.^1^^,^^2^ Hydroquinidine can be used for ventricular electrical storm with efficacy in patients with Brugada syndrome, early repolarization syndrome, short QT syndrome, and idiopathic ventricular fibrillation (VF).^3^, ^4^, ^5^, ^6^ Because of its potential proarrhythmic effects, hydroquinidine is often avoided in patients with organic heart disease and there is little data on its use in cardiomyopathies. Recently, the effectiveness of hydroquinidine in suppressing drug-refractory polymorphic events starting with short-coupled premature ventricular contractions (PVC) has been reported during the recovery phase of myocardial infarction (MI) in patients with mildly impaired left ventricular ejection fraction (LVEF).^7^ Data on use of hydroquinidine in patients with severe left ventricular systolic dysfunction are limited. Here we report a case of ventricular electrical storm during the healing phase after an acute MI with severe impairment of LVEF successfully treated with quinidine.

## Case report

**Figure 1:**
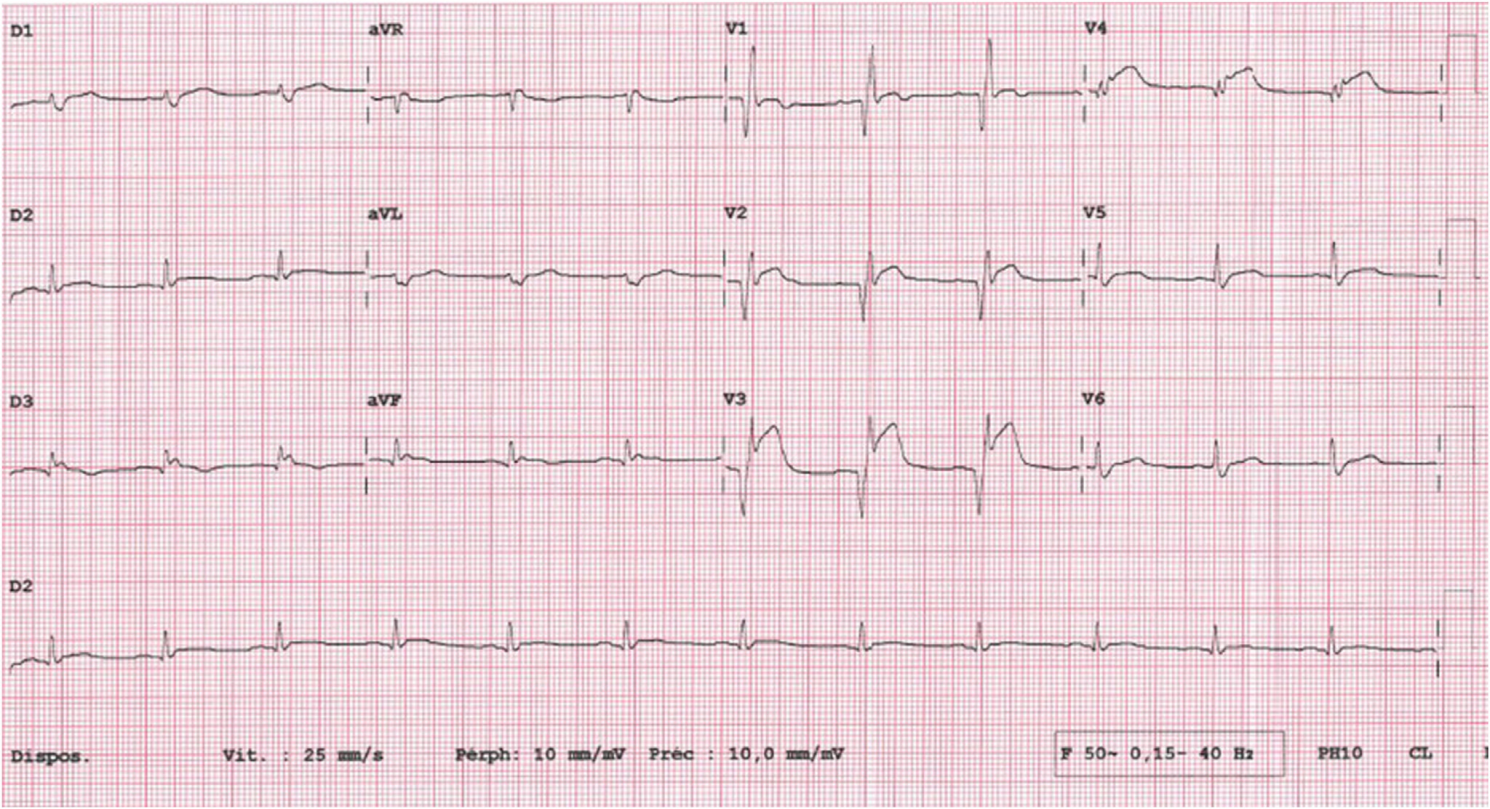
Initial 12-lead electrocardiogram showing sinus rhythm with an extended ST-segment elevation in the precordial leads corresponding to the anteroseptoapical region

A 60-year-old man with a history of smoking, Crohn’s disease, and pulmonary emphysema taking no treatment before admission called emergency 24 hours after the onset of a typical crunching chest pain. Clinical examination revealed no signs of heart failure. Initial standard electrocardiogram showed sinus rhythm with an extended ST-segment elevation in the leads corresponding to the anteroapicolateral region and the QTc of 390 ms (Figure 1). Emergency coronary angiogram revealed a total occlusion of the proximal left anterior descending coronary artery, an intermediate stenosis of the circumflex artery, and critical stenosis of the middle portion of the right coronary artery. Percutaneous coronary intervention resulted in failure of anterograde wiring crossing technique of the left anterior descending coronary artery and implantation of a drug-eluting stent in the right coronary artery. Oxygen, low-molecular-weight heparin, and dual antiplatelet therapy including aspirin and ticagrelor was initiated.

**Figure 2:**
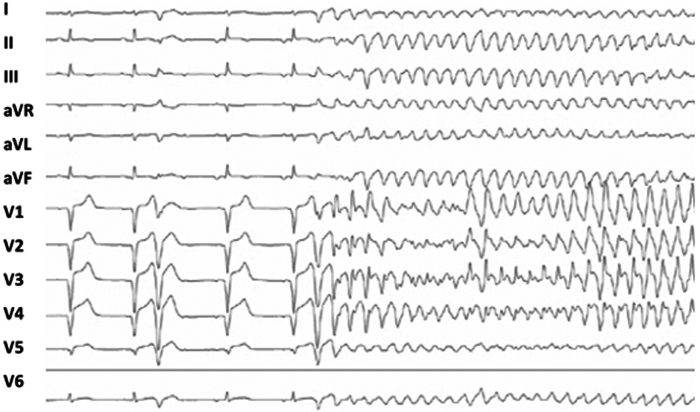
Day 5 12-lead electrocardiogram showing polymorphic ventricular tachycardia initiated by a short-coupled premature ventricular contraction manifesting negative precordial concordance in leads V_1_–V_6_, and right axis and D2-D3 discordance pointing toward the apical origin. QTc within normal limits.

On the fifth day, the patient presented a sustained polymorphic VT, recovering after 1 external electric shock, followed by recurrent similar episodes over 24 hours. All VTs showed the same pattern initiated by short-coupled PVCs and degenerating into VF. Twelve-lead recording of the PVC manifested D2-D3 discordance and right axis and negative precordial concordance in leads V_1_–V_6_ and pointing toward the apical origin (Figure 2). There were no major electrolyte disorders. Intravenous step-by-step treatment with amiodarone, magnesium sulfate, lidocaine, and oral bisoprolol was initiated. Despite rapid administration of antiarrhythmic agents, no response was observed and finally a deep sedation followed by general anesthesia were performed 24 hours later.

**Figure 3:**
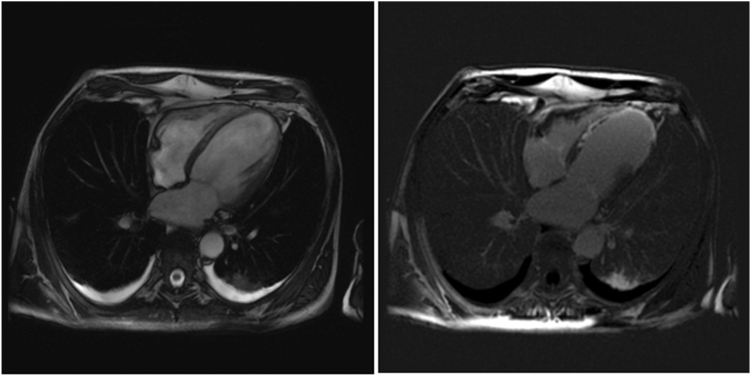
Cardiac magnetic resonance imaging (MRI). Left image: Cine MRI with myocardial edema of the septomedian and septoapical wall. Right image: Myocardial delayed enhancement of the septoapical region.

Incipient cardiogenic shock occurred and transthoracic bidimensional echocardiography showed a severe biventricular dysfunction with the LVEF of 10% using the Simpson method and the presence of apical LV thrombus. Bisoprolol was replaced with dobutamine up to 10 μg/kg/min and heparin was administered. Given the persistence of frequent recurring VT episodes initiated with the same short-coupled PVCs and sinus bradycardia 40 beats/min, a temporary ventricular pacing lead was introduced in the coronary sinus with a pacing rate programmed at 110 beats/min aiming to reduce VT episodes and improve hemodynamics. After an initial 10-hour efficacy, the patient presented VT episodes, again requiring external electric shocks. Catheter ablation of triggering PVCs being contraindicated, a left stellate ganglion blockade was performed by administering local bupivacaine bolus injection under ultrasound guidance. In the following 24 hours, malignant PVCs initiating VT recurrences reappeared as soon as the pacing rate was decreased. Given the failure of previously used conventional therapies, the implementation of mechanical hemodynamic support was discussed. Finally, we decided to introduce oral hydroquinidine with the initial daily dose of 1200 mg (600 mg twice daily). The patient continued developing short-coupled PVCs but the VTs subsequently ceased, allowing weaning from the pacing lead. Once the arrhythmias resolved, the dose of hydroquinidine was reduced to 600 mg/d (300 mg twice per day). Hemodynamics progressively improved and the dobutamine was discontinued. Bisoprolol was restarted 5 days after the introduction of hydroquinidine. The patient was extubated and a cardiac magnetic resonance imaging was performed, showing a large anteroseptoapical and lateral myocardial scar without left ventricular thrombus and an LVEF of 19% (Figure 3). There were no recurrent arrhythmias on hydroquinidine and bisoprolol, and an implantable cardioverter-defibrillator was implanted before discharge. Hydroquinidine was discontinued 10 days after discharge and no arrhythmic event was reported at 2-month visit.

## Discussion

Ventricular electrical storm, which is associated with an up to 3-fold risk of mortality, is challenging to treat and often requires a multidisciplinary coordination of medical and invasive management.^8^ Recurrent polymorphic VTs triggered by short-coupled PVCs during the healing phase following myocardial infarction presented here were refractory to conventional medical therapy and showed no response to deep sedation, cardiac pacing, and stellate ganglion blockade. At this point, catheter ablation of ventricular triggers can be considered to achieve effective VT suppression and long-term arrhythmia control.^9^ In the presented case, catheter ablation of the triggering ectopic beats could not be performed because of an intraventricular thrombus. Hypotension and myocardial stunning owing to refractory VTs, repeated shocks, and use of anesthesia can lead to consideration of a rescue cardiopulmonary support to prevent hemodynamic decompensation.^10^ However, outcome data of this intervention are lacking.

The use of hydroquinidine, considered for a long time as controversial in patients with organic cardiomyopathy, was efficient in electric ventricular storm in patients with coronary heart disease and mean LVEF >35%.^7^ However, the effect of hydroquinidine on recurrent VTs triggered by short-coupled PVCs in severely impaired systolic ventricular dysfunction with incipient cardiogenic shock has not been reported. In the presented case of a patient with refractory VTs during the recovery phase of myocardial injury, LVEF of 10%, and hemodynamic instability, the use of oral hydroquinidine allowed resolution of life-threatening ventricular arrhythmia and appeared as a rescue therapy.

The Purkinje system is supposed to have a potential role in the triggering and the perpetuation of ventricular arrhythmias in patients with channelopathy or structural heart disease.^11^ In acute MI peripheral Purkinje fibers can still remain excitable within the subendocardial border zone of scar, possibly having an oxygen supply through direct LV cavity oxygenation, and can become a driver of refractory polymorphic ventricular tachycardia.^12^ Regions of healed myocardial infarction, or completed infarction treated with surgical revascularization, are supposed to be the sources of microreentrant rhythms involving the Purkinje system or macroreentrant VT circuits generated by Purkinje cells resistant to anaerobic conditions and suffering from molecular changes. PVCs from the LV Purkinje system represent about 4% of idiopathic PVCs and half of initiating beats of idiopathic VF, and usually arise from the left fascicular system.^13^ In the presented case, the supposed apical site of origin of the PVC could be the consequence of the Purkinje system damage owing to completed infarct within the more proximal network. Quinidine was supposed to restore electrical homogeneity in Brugada syndrome by inhibiting the prominent *I*_to_ (transient outward) current in the right ventricular epicardium.^14^ Antiarrhythmic efficacy of the quinidine was also attributed to the prolongation of the ventricular refractoriness and to its anticholinergic effect.^14^^,^^15^ Molecular changes in damaged Purkinje system were reported. However, the mechanism of action of hydroquinidine in patients with acute myocardial infarction remains to be elucidated. This therapeutic option can be taken into account in similar clinical situations. However, its potential side effects should be taken into account, and particularly its negative inotropic effect, QT prolongation, and the risk of inducing bradycardia while associated with antiarrhythmic agents. For this reason, and having in mind the risk of other extracardiac adverse events, including diarrhea, cautious monitoring should follow the initial dose administration and the association with amiodarone should be avoided. More data are needed to recommend the use of hydroquinidine in patients with ischemic cardiomyopathy with severe systolic dysfunction presenting with ventricular electric storm.

## Disclosures

None.
